# Epistemic Consultants and the Regulation of Policy Knowledge in the Obama Administration

**DOI:** 10.1007/s11024-020-09411-8

**Published:** 2020-06-25

**Authors:** Jack Wright, Tiago Mata

**Affiliations:** 1 Centre for Research in the Arts, Humanities and Social Sciences, University of Cambridge, 7 West Rd, Cambridge, CB3 9DP UK; 2 Science and Technology Studies, University College London, Gower Street, London, WC1E 6BT UK

**Keywords:** Expertise, Governance, Big data, Open government, Dispersed knowledge, Behavioral economics

## Abstract

The agencies of the government of the United States of America, such as the Food and Drug Administration or the Environmental Protection Agency, intervene in American society through the collection, processing, and diffusion of information. The Presidency of Barack Obama was notable for updating and redesigning the US government’s information infrastructure. The White House enhanced mass consultation through open government and big data initiatives to evaluate policy effectiveness, and it launched new ways of communicating with the citizenry. In this essay we argue that these programs spelled out an emergent epistemology based on two assumptions: dispersed knowledge and a critique of judgment. These programs have redefined the evidence required to justify and design regulatory policy and conferred authority to a new kind of expert, which we call epistemic consultants.

The central claim of this essay is that the administration of Barack Obama adopted a picture of knowledge that disempowered experiential forms of expertise and endorsed what we call epistemic consultants. Claims to expertise may be ground on detailed knowledge and experience within domains of practice and policy, like public health or education. During the Obama administration, a set of initiatives were enacted that downgraded the authority of these actors in favor of social scientists without any attachment to a specific experiential/policy domain, but with skills that applied across government. In the place of the testimony of domain-specific experts, the design of public policy favored reliance upon data aggregation, pattern matching algorithms, behavioral experiments, and simulated and actual randomized control trials. We believe that comparable developments have taken place in other Western nations and deserve close scrutiny.

One of science studies most arresting claims is that nations exhibit cultures of rationality, or in the apt formulation of Sheila Jasanoff: “‘public knowledge ways,’ that comprise preferred modes of producing public knowledge and conducting policy deliberation” (Jasanoff 2012: 9). In this essay we argue that the picture of knowledge that emerged during the Obama administration prescribed certain knowledge ways, and we show by what institutional mechanisms, agencies, and actors these were implemented in the government of the United States of America. Science studies has tended to view national epistemic cultures as resilient and has richly described them in case studies of how risks are managed and futures imagined (Ballo 2015; Bouzarovski and Bassin 2011; Felt 2014; Fonseca and Pereira 2014; Jasanoff and Kim 2009; Jasanoff and Kim 2015; Kim 2014; Levenda et al. 2019; Tidwell and Smith 2015). These studies reveal the American civic epistemology to be “contentious” (Jasanoff 2007: chapter 10) when compared to its counterpart in France, UK, Germany, or the European Union. Our account complements these claims by showing how the character of public reason may be studied beyond the contrast between national polities (and the complex of government, courts, academies, and civil society) by looking at institutional change. But it also highlights how public knowledge ways can undergo rapid change. We draw our focus onto the redesign of the information infrastructure of the US government. We pay close attention to what these designs tell us about the Obama administration’s epistemic preferences, that while not transforming the American “contentious” civic epistemology did reconfigure who wielded power and control.

At the same time as science studies scholars document national variability in pathways to knowledge, they have also worried about the simplistic models of expert authority found in campaign materials of learned societies, such as the Royal Society of the United Kingdom or the American Association for the Advancement of Science. As organized science sets out to claim a monopoly of authority, science studies scholars counter it with pleas for public engagement and for extending our definition of expertise (Collins and Evans 2002 famously set this as *the* problem for contemporary sociology of science.) Our essay shows that the Obama administration’s implicit answer to the question “who is the expert?” matches neither the ambitions of scientist advocacy groups nor the counsel of its sociological critics. We contend that the US government came to rely on psychological and economic theories of knowledge to decide on what experts to trust.

The major initiatives we discuss in this essay were presented to the citizenry as fostering open and smart government. These initiatives changed the types of evidence that government departments and agencies were required to collect and act upon, both in what to consider when marking the decision to intervene (upstream) and in how to communicate with the citizenry (downstream). Although not explicitly codified, we hypothesize that Obama’s information reformers shared two epistemic assumptions. (i) The kind of information most relevant for assessing, creating, and enacting policy is dispersed throughout the population; it is everywhere and in everyone and requires special techniques and tools to be revealed. (ii) Human judgment is unreliable; individuals frequently make errors and exhibit biases so their judgment and reasoning should be considered with caution.

The initiatives inspired by this epistemology emerged in the course of the Obama administration. They did not come pre-formed, rather they were the incremental and idiosyncratic achievement of agencies and actors in collaboration, working from within an institutional culture. It is for this reason that we must pay special attention to a few key institutions that wielded the power to promote information management reforms during Barack Obama’s first term in office. The primary mover of these reforms was the office of the US government charged with oversight of information, the Office of Information and Regulatory Affairs (henceforth OIRA). In our first section, we briefly review the culture and track record of OIRA. We explain how from its creation the agency has acted as an instrument of an administrative presidency that compelled agencies to account for their informational practices. That vocation and function made it attractive to those seeking to use information management to change the knowledge ways of the US government. We then outline Obama’s reforms by attending to the two key aspects of the Obama administration’s epistemology ((i) and (ii)). In our second section, we focus on dispersed knowledge. We document key statements by important actors in the administration, notably, Obama’s first-term ‘Regulatory Czar’ and OIRA head, Cass Sunstein, and describe how the idea that knowledged is dispersed throughout society was used to justify open government and big data policy initiatives. In our third section, we discuss behavioral economics’ account of judgment. Our argument again examines the ideas and statements of key policy entrepreneurs to explain why they promoted empirical and behavioral evidence and viewed testimony and deliberation with skepticism. In our last section, we argue that the changes and ideas we document should be seen together and that they served to undermine the authority of experience-based forms of expertise while promoting the skills of those we call epistemic consultants.

## Regulating the Regulators

A feature of contemporary policymaking in the United States is the increasing reliance on mechanisms of administrative control. Through the Office of the Management of the Budget (OMB) and its grip on funds, the White House has means to constrain and direct regulatory agencies and programs that ought formally to answer to Congress. For our purposes the Office of Information and Regulatory Affairs (OIRA), a small subsidiary of the OMB, is of special interest since it exerts control through a grip on information.^1^ In this section we review the political context that brought OIRA into being and how its function within the American government propelled it to become an important conduit for informational reforms. We content that to a large extent these reforms build upon a pre-existing political and administrative culture.

OIRA was created by the Paperwork Reduction Act of 1980 with the assignment of overhauling federal collections of information from the public. The bipartisan bill pledged to de-bureaucratize and improve coordination between agencies, and thus ease the burdens of form filling that weighed on families and firms. The benign mandate to “cut down the red tape” followed a tradition of Federal Reporting acts that went as far back as 1942, however, the bill’s outcomes were almost immediately entangled with a distinct and more radical set of concerns, those of deregulation. OIRA would not merely simplify the communication between regulatory agencies and the public, it would become instrumental in eliminating components of that relationship.

By way of an Executive Order (#12291 of February 17, 1981) the then recently inaugurated President Ronald Reagan introduced the requirement of cost benefit analysis on new federal regulations (Reagan 1981).^2^ Government agencies were required to file “Regulatory Impact Analyses” to the Director of the OMB. OIRA was to estimate the cost-benefit appraisals, a role that under Presidents Gerald Ford and Jimmy Carter had been informally taken up by the Council of Economic Advisers (DeMuth 2011: 16).^3^ Advocates for Reagan’s review bottleneck claimed that it saved 10 billion dollars annually with an additional 9–11 billion dollars one-time savings. From 1981 to 1989 OIRA reviews returned, changed, or withdrew over 30 rulings from the departments of Health and Human Services, Education, Labor, and Housing and Urban Development (Weidenbaum 1997: 23; Miller 2011).^4^

By the later Reagan years, OIRA directors were under suspicion of consulting with business organizations and acting in their interests. In 1986 Congress threatened to drain OIRA’s funding until its analyses were released for public study. The White House complied with the demands of Congress and accepted curbs on the office’s actions, including subjecting the nomination of directors to Congressional confirmation (Harris and Milkis 1996; Morrison 1986). It was under these new rules that President George H. W. Bush was prevented from appointing a Director, and instead created an adhoc Council on Competitiveness to supervise the Office’s actions. During his administration there were few notable changes to OIRA’s functioning, and the Office kept its reputation as an agent of the Republican White House’s constraining of the regulatory state.

By contrast, in his first year at the White House Bill Clinton issued an Executive Order (#12866, September 30 1993) that encouraged regulatory interventions (Clinton 1993). Clinton established OIRA as the preeminent bureau for regulatory oversight, for planning and to an extent even for the design of rules. He charged every agency to appoint a Regulatory Policy Officer to liaise with OIRA, mandated every agency to produce a Regulatory Plan for review and compilation, and required agencies to self-examine their progress against the plans. The vision was that OIRA would centralize expertise on rulemaking; and lay out before all alternative programs of action and inaction.

During the George W. Bush Presidency allegations of industry capture briefly resurfaced, with the press reporting that industry think tanks were liaising with OIRA to create “hit lists” for deregulation. But despite these concerns and Bush’s record of deregulation being longer than Clinton’s, there was no return to OIRA as regulation buster (Eisner et al. 2006; Power and Schlesinger 2002). The regime that the Obama administration inherited was, thus, the one remade by the Clinton executive order and by the rewriting of the Paperwork Reduction Act in May 1995. That legislation asserted Presidential control over the independent agencies, and through its influence on agencies’ rulemaking extended the discretion of the administration (Kagan 2001). Although a small office with few staff, OIRA came to wield “outsize power” by coupling its status as regulator of regulators to its stewardship over the information policy of the administration (Weisman and Bravin 2009).

Obama and his advisors saw control of rulemaking and information through OIRA as a valuable instrument for an administrative presidency. To head the office, Obama appointed his former colleague at the University of Chicago Law School, Cass Sunstein. Sunstein had long trumpeted information management as a significant and underutilized instrument of government (Sunstein 1986, 2005; Sunstein et al. 1998). OIRA’s history as information regulator of all other agencies led Sunstein to see it as the ideal venue from which to change the government’s culture of information and evidence use.^5^

As we will describe, Sunstein used the ideas that knowledge is dispersed in society and that individual judgment is often deficient to justify initiatives aimed at opening the government’s store of information and at tailoring policy and communication as “nudges” to public behavior and the behavior of policy makers. Obama reframed OIRA in an executive order that bears the imprint of Sunstein’s vision (#13563, January 18 2011), issued just after the Democratic Party lost its majority in the House of Representatives, i.e. at a moment when an administrative presidency became an inevitability. It set out to move OIRA from the juncture of regulatory agencies and the White House to being the point of interface between government, agencies, the scientific community, and the public and to become the compiler of their collective wisdom (Obama 2011). Whilst under Clinton, OIRA would interact with agencies behind closed doors and then publicize its decisions and reasoning, under Obama OIRA invited the public to contribute to its reviews at an early stage and to “comment through the Internet on any proposed regulation.”^6^

In 1980 OIRA was created to be a gatekeeper that would curtail requests of information by agencies and thus relieve the public from government’s surveillance. OIRA made its reach felt across the Mall as impediment, imposing cost-benefit reckonings on agencies’ actions. In the early 2010s OIRA was reinvented as the gateway through which the public would flood the halls of government with comment, direction, and insight. Despite a rhetoric of openness, Obama-era OIRA’s gateway of consultation and knowledge exchange did not admit entry to all. As we describe below, OIRA intruded into rulemaking by imposing to agencies new standards of evidence—including retrospective evaluation, experimentation, and data analytics. These new standards were justified as privileging a more robust set of knowledge ways touted as “data-driven, evidence-based regulation and to select approaches on the basis of empirical findings, rather than intuition, anecdote, or guesswork” (OMB 2010). OIRA’s presentation before the agencies it oversaw was that of an epistemic authority, a knowledge overseer.

## Dispersed Knowledge

At the time of Obama’s election, media reports and political pundits criticized the American government’s lack of regard for science advice. The Union of Concerned Scientists had since 2004 waged a campaign against President George W. Bush’s “unprecedented” “manipulation, suppression and misrepresentation of science.” (2) The Bush administration’s handling of scientific advice created a scandal in June 2003 when it was revealed that the White House had edited a report by the Environmental Protection Agency on climate change. In its protest the Union of Concerned Scientists built a case setting climate change denial alongside censorship of research in reproductive health, air quality, and endangered species and listed numerous appointments of dubious merit to scientific advisory bodies. “Scientific integrity” was in peril because of the choice of unqualified and compromised advisors (Shulman 2008; Abraham and Ballinger 2012).

Barack Obama pledged to repair science advice. In a memo of March 9 of 2009, two months after his inauguration, he instructed heads of agencies and executive departments to maintain “the highest level of integrity in all aspects of the executive branch’s involvement with scientific and technological processes” (Obama 2009a). Strikingly, it took 20 months before procedures implementing “scientific integrity” were set in place, including nominating the Union of Concerned Scientists’ scientific integrity officer to scrutinize allegations of tampering with government science. These responses suggest that “integrity” and channeling advice from organized science was not a high and urgent priority of the administration. A very different kind of epistemological attitude drew greater emphasis and it did not, primarily, call upon credentialed scientists.

In one of his early speeches as head of OIRA, Sunstein (2010c: 19) explained that the administration was trying to “ensure that institutions benefit from the dispersed knowledge of Americans.”^7^ OIRA’s reports to Congress between 2009 and 2015 regularly repeated the idea that “knowledge is widely dispersed in society” (OIRA 2009: 39). The President himself adopted that phrase. When remarking that “information maintained by the Federal Government is a national asset,” Obama added that “[k]nowledge is widely dispersed in society, and public officials benefit from having access to that dispersed knowledge” (Obama 2009b).

In some of his speeches as a government official and in most of his earlier publications, Sunstein explained “dispersed knowledge” by referring to a famous essay of 1945 by Austrian economist and political philosopher, Friedrich von Hayek, entitled “The Use of Knowledge in Society.” Despite similarities, Hayek’s and Sunstein’s conceptions of dispersed knowledge differ in some important ways. Hayek was writing to a debate about the possibilities of planning. To counter the socialist project of codifying knowledge and calculating the rational course of action, Hayek argued that the knowledge relevant for planning was intrinsically personal and dispersed throughout society. According to Hayek, the unstable and sometimes inarticulable nature of this knowledge prevented it from being collated by any supra-individual entity. Instead the market and the price mechanism, which Hayek believed emerged spontaneously and without any higher design, was the best way to set personal knowledge in motion and concert. This developed into the neoliberal view that market-like structures and prices should be adopted for all social interactions that require the coordination of information (Mirowski and Nik-Khah 2017; Mirowski and Plewhe 2009).

For Hayek’s purposes the distinction between knowers and non-knowers was not salient. The knowledge he was concerned with was knowledge of wants, desires, and specific circumstances, which one assumes all individuals possess in some measure. Obama, Sunstein, and their collaborators, on the other hand, sought to harvest a different variety of knowledge. As well as wants, desires, and circumstances, government agencies need to make use of specialist and technical knowledge. But, because such knowledge is asymmetrically apportioned, tapping it requires cunning strategies of elicitation, assembly, and processing. Sunstein’s argument was that adopting Hayek’s outlook on economic knowledge would provide appropriate strategies to manage this specialist knowledge. The extent to which people trust what they think they know was assumed to correlate with the extent to which they are willing to act (or bet) on it. Sunstein’s claim was that the way to harvest the specialist knowledge he was concerned with is to aggregate people’s actions in a free entry and exit environment where knowledge is rewarded (either monetarily, with influence, or by other means), not unlike a market. In the 2006 book *Infotopia*, Sunstein discussed wikis and open source software (online collaborations that anyone can contribute to and edit) and prediction markets (where predictions about events are garnered from bets) as examples of market-like processes for aggregating specialist and technical knowledge. The idea is that if enough people have access to the source code (for software), editable page (for a wiki), or betting counter (for a prediction market), then their partial knowledge will combine to debug programs (software), correct errors of fact (wiki), or anticipate the future (prediction markets) (Raymond 2000).^8^

The handling of these systems of information elicitation and aggregation do not typically require specialist knowledge of the issues under consideration. However, they do require management and oversight by a specific kind of actor that is familiar with the digital platforms and knows how to design information architectures that encourage participation. Unlike Hayek’s markets there is not the assumption of a spontaneous order.

Several flagship initiatives of the Obama Presidency were conceived as exercises in knowledge aggregation. One of Obama’s first actions as President was to release a memorandum on Transparency and Open Government.^9^ Open government was more expansive in its ambitions than transparency, promising three types of benefits: the disclosure of information would improve markets by leading to more informed decisions (Sunstein 2011); datasets would be created for empirical work on how to improve policy; and, open government would encourage the crowd sourcing and aggregation of insights and innovations across government. It is with this horizon that in April 2009 the OMB launched data.gov, a centralized repository for all government data, and whitehouse.gov/open, a website designed to enable the meeting of “government employees with the knowledge and know-how of the American people” (Office of the Press Secretary 2009).^10^ As part of the open government program departments were directed to improve and increase the collection of information, publish it online, and institutionalize cultures of collaborative government (Orszag 2009a). OIRA took a leading role in directing the roll out of the various parts of this initiative and created its own online resource where the public might follow the regulatory reviews undertaken by its staff, reginfo.gov.^11^^,^^12^

The epistemic aspirations of the open government initiative became more explicit when it was reframed as a big data project.^13^ The released government information would from thereon (March 2012) be in a machine-readable format, a GitHub repository of resources and tools was created to accelerate open data use, and $200 million was committed to improve state computing and data analysis infrastructure. It was now also hoped that digital data analysis would reveal government success and failure on the cheap (Krueger 2012). The expectation was that data could be analyzed through emerging pattern finding algorithms to unearth useful ‘insights,’ or that it could be used to simulate randomized control trials. Data was released in formats easy to harvest, alongside open-source tools, so that those with knowledge of information extraction and processing might study the data and pass insights back to the government.^14^ Through hackathons on challenge.gov, for example, the government sought to “engage citizens in solving difficult problems” by reimagining those problems as data analysis competitions (Open Government Partnership 2013). Teams of analysts were invited to download datasets and search for patterns within them in order to come up with solutions to the pre-specified problems.^15^ The initiatives were welcomed by data analytics firms. Google’s chief economist, Hal Varian (2010), commented that such large-scale data analysis would provide an alternative to paying for the services of expertise. The open invitation for citizen engagement therefore in practice narrowed into appeals for data scientists and firms to study the records of the US government. To understand why collaborative knowledge making took this form we need to look at the second key dimension of this emerging epistemology.

## Critique of Judgment

In addition to the vision of dispersed knowledge, Sunstein and other members of Obama’s two administrations, like OMB director Peter Orszag, subscribed to the critique of individual cognition emerging from behavioral economics.^16^ The seminal contributions that anchor behavioral economics came from two Israeli experimental psychologists, Amos Tversky and Daniel Kahneman, collaborating in the study of cognition and subjective probability. By the early 1970s they had identified a set of routines, or heuristics, that aided but also warped individuals’ understanding of information.^17^ Although not usually acknowledged, Tversky and Kahneman were moved by a suspicion of, what we today might call experiential expertise, the authority of the judgments of medical doctors, professors of statistics, and other formidable experts (Lewis 2016). Tversky and Kahneman never committed to a mechanism explaining the shortcomings of cognition, but in recent years their followers, and Kahneman himself (2012), have endorsed a dual model of the brain. In Dr. Jekyll and Mr. Hyde fashion, a “system one” that is impulsive and emotional is believed to inhabit the limbic regions of the brain. By contrast, a system “two” for more demanding calculus and reasoning is associated with the posterior (prefrontal) areas of the cortex (Sunstein 2013a: 204). Behavioral economics warns of an imminent threat of unreason that is wired to the human neural architecture. The normative program of behavioral economics is to set straight individual choice, either by strengthening system two, or by correcting the wrongs perpetuated by system one such as a lack of self-control, short-termism and various biases in judgment (the byproducts of the mentioned heuristics).

Sunstein and his longtime collaborator, the 2017 winner of the Economics Nobel memorial prize, Richard Thaler, have argued that regulators must take advantage of “the emerging science of choice.” They believe that individuals can be steered towards rational outcomes, by which they mean the choices individuals would have made if they had self-control, were fully informed, and had the time and cognitive resources to calculate the best course of action. Through default rules and other “nudges,” governments and the private sector should “make it automatic” and thus “ensure that if people do nothing at all, things will go well for them” (Sunstein 2006: 100).^18^ A key 2010 memo by Sunstein on ‘Disclosure and Simplification as Regulatory Tools,’ suggested multiple ways information could be structured to get the right messages across, and how the disclosure of information could constitute nudges. The memo distinguished between “making a merely technical disclosure—that is, making information available somewhere and in some form, regardless of its usefulness—and actually informing choices” (Sunstein 2010b). The dull work of disclosure could become a “low-cost, high-impact regulatory tool” (Sunstein 2010c, d) provided it is “preceded by a careful analysis of their likely effects” (Sunstein 2010b).

Enthusiasm for behavioral science continued even after Sunstein and Orszag left government, and in fact seems to have blossomed after their departure. In the final years of the Presidency, Obama issued an executive order “Using Behavioral Science Insights to Better Serve the American People” (#13707, September 15, 2015), that encouraged agencies to draw on “research findings from fields such as behavioral economics and psychology about how people make decisions and act on them.” (Obama 2015) The executive order created a Social and Behavioral Sciences Team (SBST) within the West Wing to advise on behavioral approaches to communication and choice architecture. The team was part of the Office of Science and Technology but openly collaborated with OIRA.

One of Kahneman and Tversky’s powerful insights (although not exclusive to their approach) was that they could manipulate individuals’ decisions by tweaking the presentation of the problem. They never contended that they could transform the human subject, who was always deemed immutable and a black box. The work of psychology was focused on the choice not on the chooser. Kahneman’s research in vision (for a later instance, see Kahneman and Henik (1981)) reveals an interest for gestalt psychology (Heukelom 2014: 107–111). Perceptual illusions were a frequent metaphor in presentations of ‘prospect theory’ (see in particular Kahneman and Tversky 1986).

The direction of actors’ attention is a crucial aspect of nudging and is evident in numerous behavioral inspired initiatives of the Obama Presidency. In its report to Congress in 2009, OIRA explained that the policy payback of behavioral economics was “simplifying choices through sensible default rules and reduced complexity” and “by increasing the salience of certain factors and variables” (OIRA 2009: 37). Many of the interventions inspired by behavioral economics are visually labored to seize attention and direct action. The team of young postgraduates that made up SBST claimed early success in improving outcomes with Service members’ savings, low-income students’ enrolment in colleges and loans, and enrolments in health insurance (SBST 2015). The interventions comprised of phrasing and visual presentations of emails, orientation briefings, notices, reminders, and personalized letters. The government’s reach over disclosure and information requirements and its significant communicative power offered it an opportunity to redesign choice situations.

**Fig. 1:**
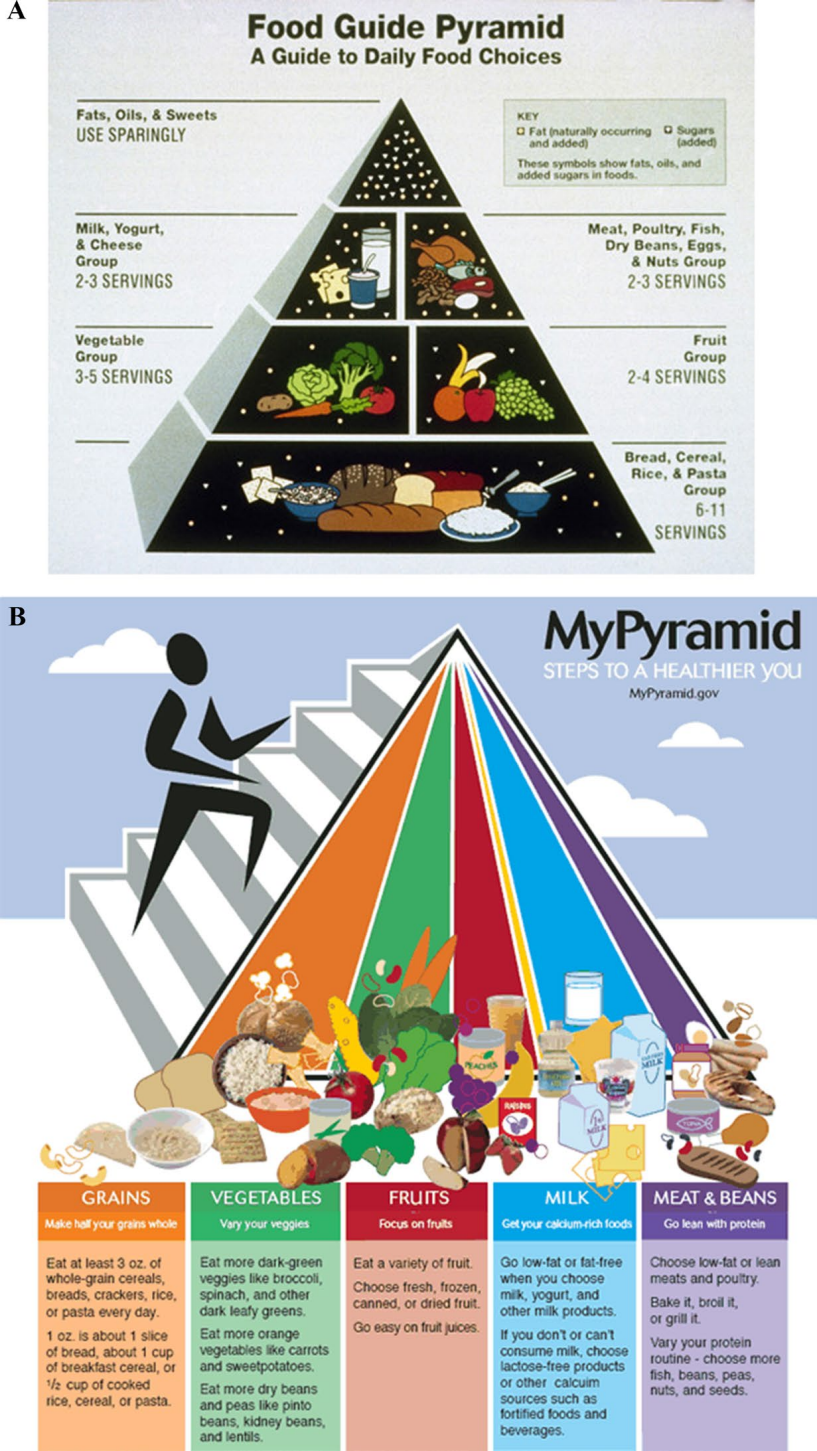

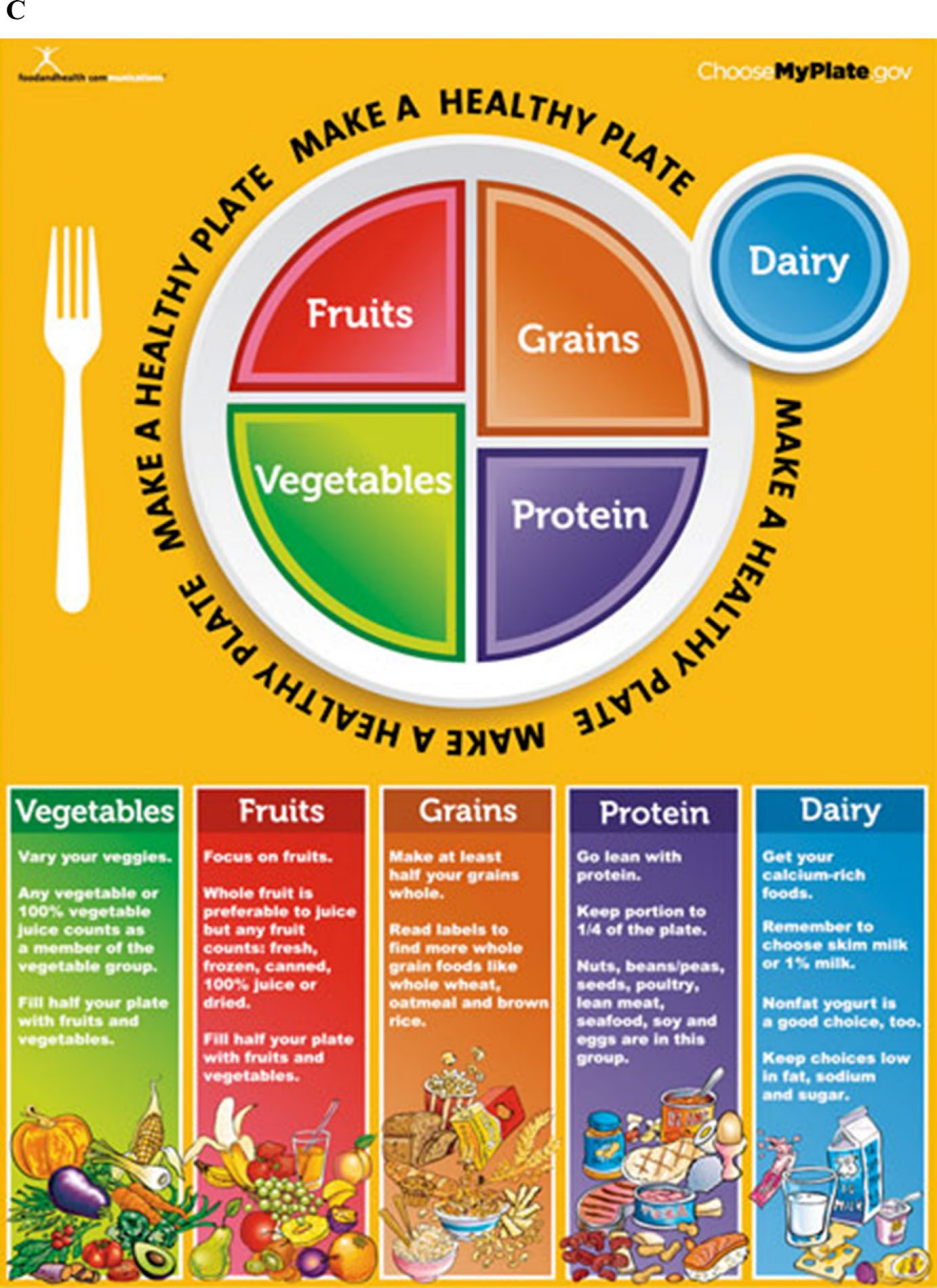
**A** The Food Pyramid of 1992. **B** The MyPyramid. **C** MyPlate in 2011

The part played by behavioral science in regulatory design is well displayed in the case of MyPlate. In 1992, the United States Department of Agriculture (USDA) introduced the Food Guide Pyramid that illustrated the elements of a healthy diet.^19^ To update the underlying nutritional information, the USDA retired the Food Guide Pyramid in 2005 and replaced it with MyPyramid. However, the new presentation was seen by many as confusing and in June 2011 the USDA introduced MyPlate (see figures 1A–C). Each change followed a process of advanced study (OIRA 2009: 37). The pyramid representation gave citizen gourmands a measure of the shares of foodstuffs by way of a hierarchy. Its legibility was potentially problematic because it placed the least healthy foods at its summit (Fig 1A). The later version sought to preserve the familiarity of the pyramid without the ranking, setting out the shares of each food group in a healthy diet (Fig 1B). The plate took from the pyramid the idea of color-coded shares but placed on a pie chart that by recalling, by name and likeness, a plate invites citizens to compare their meals to the icon.^20^ The benchmarking of meal and advice is made as immediate as it can be. As the First Lady explained at its unveiling, moms and dads are too taxed with time to be also nutritionists but “we do have time to take a look at our kids’ plates. As long as they’re half full of fruits and vegetables, and paired with lean proteins, whole grains and low-fat dairy, we’re golden. That’s how easy it is” (USDA Office of Communications 2011).^21^

MyPlate is appealing in its parsimony and immediacy, quickening decision time, unburdening individuals of reading through long explanations.^22^ It is kind on the time and attention of citizens. The idealized choice environments induce individuals towards the right choices without need for elaborate judgment, calculation, or attention. These designs, rich in visual metaphor, nearly always abstract from and hide away the reasoning behind the guidance. The solution is not to make a better human brain nor to impel individuals to act slowly and thoughtfully. The ideal is to create environments where the right choices are nearly automatic (Schüll and Zaloom 2011).

Liberal political theory assumes that individuals will know themselves and act in agreement with their circumstances and desires, as sovereign of their choices. After the lessons of Tversky and Kahneman it seems reckless to trust individuals’ better judgment. Behavioral experiments reveal that even sophisticated, educated persons give inconsistent accounts of their wants and preferences and engage in faulty reasoning. The matter is worse when individuals form choice collectives and meet to deliberate, because all sorts of bad interactions such as informational and reputational cascades are said to ensue and confound the best course of action (Sunstein and Hastie 2014). If one accepts behavioral economics’ skepticism that actors’ may not always command their own reasons or judge situations wisely, then mechanisms aiming to elicit fruitful collaboration and knowledge exchange need to protect actors against the appeal of bias and error or reckon those failings into the analysis. The open government initiative (and the subsequent big data initiative) were designed with this in mind. In Whitehouse.gov/open and data.gov the stress was on modes of participation that were atomistic and depersonalized, erased of social markers and opportunities for sociability. The public was believed smarter under a shroud of anonymity and facing the glare of a computer screen. Other elements of the open government initiative did away with deliberation completely. By reimagining social issues as data analysis competitions, the hackathons of challenge.gov, for example, removed any form of deliberation from the determination of policy interventions. The frustrating message of behavioral economics about the limits of human judgment, thus validated the algorithmic rationality promised by the big data initiative. Obama’s big data and open government initiatives, in turn, elevated the standing of behavioral readings of data and evidence by building data sets on choice and emphasizing empiricism (both pattern matching and simulated and real randomized control trials).^23^

The social science of choice applies not only to householders but also to the government and its advisors. Behavioral economists distrust the wisdom of fast thinking experts, whose authority derives from experience (for a contrasting standpoint that values heuristics, see Gigerenzer 2008). Behavioral economics levels the authority of experienced or not, credentialed or not, because the human brain will always be an unreliable interpreter of choices that involve uncertainty, self-control, future outcomes, which comes to be nearly all of social life. Behavioral economics has thus much to say about valid and invalid knowledge ways. Within the Obama administration this combined with the dispersed conception of knowledge outlined above to support a reimagining of expertise.

## Epistemic Consultants

We have described how in the last 40 years oversight over the flow of information emerged as a feature of the White House’s administrative control over the breadth of the US government. The Obama administration saw in the management of information a means to tap dispersed knowledge in the polity and to encourage the use of behavioral social science in policy design. We have argued that Obama’s informational reforms should be seen together as part of an emergent epistemology. The various open government, big data, and behavioral policy initiatives emerged in concert, congratulating the public as a privileged interlocutor and promising smarter government. They acted on the same aspect of government (the management of information), they did so through the same set of institutions (the OMB, OIRA), and they were supported by the same set of actors. Moreover, the different initiatives drew support from one another and from two key epistemic assumptions: (i) that the kind of knowledge relevant for policy is best thought of as dispersed through society; and, (ii) that individual judgment is unreliable. In this section we discuss how this emerging epistemology answered the question: who are the experts that we can rely upon to guide government policy?

The promotion of transparency in the open government and big data initiatives was in reality a project for deepening informational control. In its infancy, OIRA brought the regulatory agencies to account by way of cost-benefit calculations. In the Obama years, oversight of regulatory processes was established by translating actions, past, present, and future, into machine-readable data that could be coded, stored, monitored and analyzed digitally. Traditional procedures of consultation such as focus groups appeared to these open government champions as “often highly artificial” and “sometimes test what people like rather than what they would actually do” (Sunstein 2013a: 188). Instead, people’s behavior—what they do and how much they do of it, not their judgments and how they reason them—was emphasised as the input of analysis. Although transparency appeared as a democratic pledge, of the elected addressing the electors, in practice it called into being systems of standardization and evaluation that asserted the command of the White House over the procedures of regulatory agencies.

Experiential expertise—with detailed knowledge of particular processes, environments, and capacities accumulating through experience—was undermined by the idea of dispersed knowledge. In its place OIRA elevated information aggregation, data analysis, and randomized control trials as archetypes of evidence for policy. These archetypes became increasing emphasized as ways of cutting through the supposedly blinkered conversations on policy issues like health and education and getting at “what works” (Orszag 2009b, c). Experienced domain specific experts were supplanted by *epistemic consultants* who could aggregate and process information from a variety of sources in cunning ways that typically did not call upon introspection, individual testimony, or debate.

The promotion of behavioral policy followed a similar pattern. The critique of judgment within behavioral economics raises doubts over what experts can claim to know. But, while behavioral economics is critical of expert judgment it does not call for a removal of scholars from public service. As with information collation procedures, the design of choice architecture calls for skills that are not bound to any domain of policy or human action. The recruitment of domain specific experts with on the ground knowledge, who might tailor interventions to social context, was replaced by data analysis and policy experimentation from experts attuned to the faults of human cognition and by interventions that circumvented judgment and streamlined decision.

Like those called on to aggregate information and look for insights in government data, the Obama wiz kids at the SBST had no subject specific competency (such as education, defense, or welfare). They brought to government a general competency in evaluating evidence on the effectiveness of alternative regulations and a new approach to the design of interventions. Rather than deferring to those with knowledge of particular domains (inside or outside government) or convening stakeholders in focus groups to identify what disclosure regime would work best in food labeling, or in credit card statements, the regulator should seek the guidance of randomized control trials and behavioral experiments (Orszag 2009b, c).^24^

The information reforms of the Obama administration drew no grand reordering of the functions of government, for expansion or for contraction, yet epistemic consultants were placed to make contributions to every branch of the regulatory state. They were privileged guides through the vast landscape of government and behavioral data setting out opportunities for policy intervention and closing off futile regulation. But because they conceive society as a system of designed choice situations, private and public, intended or not, they have also become crucial participants in the design (and testing) of new rules.^25^ They are consultants not only in the immediate sense of being hired experts advising government, they are also consultants to the citizenry designing labels, information campaigns, disclosure requirements, and government communications that guide individuals to better outcomes.

## Conclusion

We have argued that key reforms of the Obama administration spelled out a new vision for the use of knowledge in policy, based on a coupling of the idea of dispersed knowledge with behavioral economics’ critique of judgment. In practice this new vision amounted to a new set of “knowledge ways” for the US government that shifted the “preferred modes of producing public knowledge and conducting policy deliberation” in favor of information aggregation, data analysis, behavioral experiments, and randomized control trials.

The coupling of dispersed knowledge and a critique of judgment was neither spontaneous nor inevitable. From the standpoint of the history of ideas and the sociology of knowledge, the two strands have developed in academic and public spheres with no noticeable convergence. Although our goal in this paper was to describe an emerging epistemology, not to offer a definitive account of why it came into being, political and institutional circumstances might provide a plausible causal story for the coupling of dispersed knowledge and behavioral economics. Such a story could stress how an obstructionist Congress encouraged Obama officials to do policy by executive order and to deploy tools of administrative control over the independent agencies. Obama officials explicitly understood information engineering as a means to audit and command regulatory policy. The encouragement towards an administrative presidency was pursued in an institutional infrastructure that recommended the epistemic emphasis. As we discussed, throughout the 1980s and 1990s previous administrations had placed the OMB and OIRA as overseers of regulation. Rather than yield their power through the distribution of funds or other heavy-handed politics, the Obama administration sought to control through the soft power of redesigning the flow of information and centralizing the assessment of evidence. OIRA established its authority over the domain specific agencies (and their in-house or consulting experts) as an epistemic broker with outspoken preferences and guidelines.

Our story and its configuration of actors, interests, and preferences is not a full account of the Obama record, nor do we claim this to be the most salient feature of that record. Moral suasion was as important for Obama’s leadership as was bureaucratic innovation. Obama made use of his charisma and popularity to work social change by mass persuasion and moral example.^26^ At the same time, any account of the Obama presidency should not ignore that alongside his compellingly personal, affective leadership, was an austerely empiricist approach to regulatory policymaking.

The new information management initiatives were at their launch portrayed as aiming to enhance transparency and trust in government. While the American regulatory state pillared on open data and experimental social science may be more accessible, as a database of reports and spreadsheets, to anyone with a computer and an internet connection, it was not made more assessable. Policy decisions remained as opaque from the citizenry as they were before since without key new skills—in data analysis, experimental social science, and behavioral economics—the wealth of data, its meaning, and insights lay inert and incommensurable. With the motto of dispersed knowledge, OIRA and the OMB issued a call for citizen engagement. While the idea of dispersed knowledge deflated the authority of experiential experts, that authority was not passed to citizens at large. Instead, those that benefitted most were individuals and organizations that knew how to aggregate and manipulate data from multiple sources and parse that data through the grid of new ideas about cognition and choice architecture. These symbol manipulators emerged as epistemic consultants, aids to new policy evaluation and design, and a new kind of policy oversight.

One feature of the ongoing debate about the status of expertise in liberal democracies is to see the election of Donald Trump in the USA and the Brexit vote in the UK as a watershed moment for a shift in attitudes towards expert guidance. Some, mainly in the media, have called our times a “post-truth era” where sentiment is more powerful than argument, and facts are routinely muddled by conspiracy theories and hyperbole. Science studies has vigorously responded to these developments. Some eminent scholars have suggested that analysis from the discipline may have aided public skepticism and confusion about whom to trust as expert. The claim (see Collins et al. 2017; Fuller 2016; Lynch 2017; and Sismondo 2017a, b for different takes on this argument) is that by endorsing “symmetry” between beliefs (true and not true, credentialed and uncredentialled) the sociology of knowledge has leveled the epistemic playing field and has legitimated a version of epistemic democracy that empowers populists, bigots, and charlatans. To mend the damage done some authors have set out an agenda of drawing “our scientific understanding of science and expertise” to adjudicate between institutional arrangements and robust grounds of expertise (Collins et al. 2017).

Our study shows that another project of adjudicating expertise preceded alarm over a “post-truth era” and that it was successful in shaping key reforms in the government of the USA. These competitors to science studies grounded their claims not on ethnographies of knowledge making and on the study of the form of life of science, rather they launched their program with a foot in cognitive science and another in data science. Given the policy achievements outlined in this paper, the key phrases in any causal story that connects intellectuals to distrust in experts are more likely be dispersed knowledge or cognitive bias than “symmetry.” Our study shows that dispersed knowledge and behavioral inspired public policy broadcasted a deeply skeptical view of experts, many times seeking to denounce their hubris and arrogance, and that it complimented the public as knowledgeable in a very diffuse sense, particularly when interacting in online and social media.

Binaries between the power of the elites of science and the inclusion of the lay, technocracy or democracy, are confounded in the actual politics of the initiatives we describe. Public engagement through open government has given no voice to the lay, or entrusted it with epistemic authority and autonomy. Through skepticism about human judgment the epistemic consultants became the indispensable aggregators and processors of the “dispersed knowledge of the American public” (Sunstein 2010d).^27^ Behavioral governance is by design both inclusive and technocratic.

The model of epistemic governing we describe in this paper has since been dismantled because a new occupant has moved into the White House. In the view of most commentators, President Donald Trump’s approach to regulatory policy is to dismantle it. This existential threat is expressed by his choices of nominations to the Supreme Court, and nominations to heads of agencies. Nonetheless, we believe that the developments of the Obama administration should not be seen as relics of the pre-post-truth era. The legacy of that time has not been erased. In important ways what is often referred to as the “distrust of experts” is a legacy of that time. Although Sunstein and Obama’s other information reformers are often described as technocrats, their version of technocracy, relying on information aggregation and behavioral experiments, enabled a move against more experiential forms of expertise. The model of governing through information and epistemic oversight trialed in the Obama years, thus, deserves scholarly scrutiny as an important precursor to the present moment and as case of public reason in the making.^28^

## Footnotes

1 The primary sources of this study are the annual reports and support documentation issued by OIRA and various speeches and letters by its officials, among which Sunstein was the most visible and forceful. During the early stages of research for this essay, in 2015, much of this documentation was readily available online. Since 2016 and the handover to the Trump administration much of it has disappeared. The documents we examine herein are public record, we refer to the original in the form we found them and as we stored them offline.

2 According to Murray Weidenbaum (1997), the modern review process of regulation was initiated under the Presidency of Richard Nixon. For a slightly longer review, see Tozzi (2011).

3 OIRA’s role was confirmed by executive order #12498 of January 4, 1985, making the OMB the final arbiter of the regulatory process (Reagan 1985). Cost-benefit analysis became a decisive test and no longer merely consultive.

4 The most controversial single action of Reagan’s OIRA was in its dealings with the Environmental Protection Agency. OIRA, deeming market mechanisms as more robust than a ban on emissions, proposed to the EPA a marketable permits program that allowed petroleum firms with less refining capacity to purchase credits against lead restriction from those with greater capacity (DeMuth 2011: 19). With no executive discretion OIRA was an advocate for the neoliberal proposals that became emblematic of Reagan’s administration.

5 Early on in their relationship, Sunstein’s wife—Harvard Political Scientist and ambassador to the UN during Obama’s presidency—Samantha Power asked Sunstein if he ever fantasized about doing another job. She recalls his response: “I expected him to say he dreamed of playing for the Red Sox…. [Instead,] his eyes got real big and he said: ‘Ooh! OIRA!’” (Wallace-Wells 2010).

6 On May 10, 2012, Obama’s executive order “Identifying and reducing regulatory burdens” committed the White House to periodic review of regulations together with the “open exchange of information” (Obama 2012).

7 Sunstein (2010a) explains that “[f]or Hayek, a key problem is how to incorporate that unorganized and dispersed knowledge. That problem cannot possibly be solved by any particular person or board.”

8 In addition to the Hayekian idea of markets as the ideal aggregators of dispersed knowledge, this also drew on another epistemic argument: that certain forms of democracy are epistemically superior to other forms of social decision making. This epistemic argument for democracy holds that certain ways of eliciting opinions from everyone are more likely to get at the truth than the deliberations of one person or a small group of people (Landemore 2012, 2014; Estlund 2009).

9 The motto of transparency was one that Obama had adopted much earlier. In his brief time as Senator, Obama collaborated with “one of the most conservative Republicans” Tom Coburn, future presidential opponent John McCain, and fellow Democrat Tom Carper on one of his “proudest accomplishments”: the 2006 Federal Funding Accountability and Transparency Act, which set up an internet database of government spending—“USAspending” (Lehrer et al. 2008; Sunstein 2008). In the memorandum on ‘Transparency and Open Government’ Obama declared that his government would seek to: “[E]stablish a system of transparency, public participation, and collaboration… [to] promote efficiency and effectiveness in Government… [By] disclos[ing] information rapidly in forms that the public can readily find… [and by directing departments to] use innovative tools, methods, and systems to cooperate among themselves, across all levels of Government, and with non-profit organizations, businesses, and individuals in the private sector” (Obama 2009b). As we argue, the current initiatives aim at more than accountability, hinted by the second part of the quote.

10 The website regulations.gov was created during the Bush Presidency as part of its eRulemaking program. The motivation was not “open government” but rather replacement of an expensive paper-based system by a fully electronic one (OIRA 2008: 38).

11 Despite being issued before Sunstein’s nomination was confirmed, the open government project bore a significant imprint of the ideas presented in *Infotopia*.

12 For archived links to all the websites discussed in this paragraph, see (accessed December 2016): https://web.archive.org/web/20161230184053/https://www.data.gov/ https://web.archive.org/web/20161229193151/https://www.challenge.gov/list/ https://web.archive.org/web/20161231081206/https://www.regulations.gov/ https://web.archive.org/web/20161231003758/whitehouse.gov/open https://web.archive.org/web/20161229201415/https://www.reginfo.gov/public/.

13 See Office of Science and Technology Policy (2012) for an archived version of the big data initiative announcement.

14 See, for example, the “green button” and “blue button” initiatives, accessed at: https://www.whitehouse.gov/blog/2012/03/22/green-button-giving-millions-americans-better-handle-energy-costs, on February 19, 2017. Or, more recently, “The White House Open Data Innovation Summit and Solutions Showcase”, accessed at: https://www.data.gov/event/white-house-open-data-innovation-summit/, on February 19, 2017.

15 A recent example was the “Opioid Code-a-Thon” of November last year, in which “Participants will use a variety of provided datasets related to opioids and develop a solution to address one of the following challenge tracks: Opioid Treatment, Opioid Usage, Opioid Misuse Prevention.” https://www.challenge.gov/challenge/hhs-opioid-code-a-thon/, accessed 20 May 2020.

16 Despite an early career enthusiasm, Sunstein was unconvinced by the rational agent-based models of human agency that dominated the classic Law and Economics school. He instead favored empirically informed behavioral models drawn from psychology, calling such models “the most exciting intellectual development[s] of my lifetime” (Wallace-Wells 2010). With his co-authors he outlined this behavioral approach to economics and the law in a number of papers and in the popular book *Nudge* (Sunstein et al. 1998; Sunstein and Thaler 2003a, b, 2008; Sunstein 2011). Orzsag was similarly impressed stating that behavioral economics is "one of the most important intellectual developments of the past several years” (Orszag 2009d).

17 The principal heuristics were representation, availability, and anchoring (Tversky and Kahneman 1974). Repurposing some of their earlier work, Kahneman and Tversky (1979, 1981, 1986) outlined a “prospect theory” that accounted for the incoherence and inconsistency of behavior (or more technically the violation of principles of dominance and invariance). The relabeling, however, did not fundamentally change the character of their research.

18 In the book revisiting his years in government, Sunstein (2013a: 35) also adds that “we need choice architecture for choice architects,” cognitive regulation of the regulators, and that “cost-benefit analysis is a nudge” to make the government use system 2, the rational decision-maker. The choice architecture that presses the government to rational calculation is the OMB and OIRA.

19 For a history of how nutritional advice and product labels became part of the states ‘informational infrastructure,’ see Frohlich (2017).

20 For a challenge to the advice contained in the simplified plate, see https://www.hsph.harvard.edu/nutritionsource/healthy-eating-plate-vs-usda-myplate/, accessed November 24 2016.

21 In the case of fuel economy labels in cars, immediacy was achieved by setting alongside the estimate of average consumption per mile also a 5-year monetary estimate of savings from fuel efficiency, over the average vehicle. Environmental considerations were coupled with financial consideration of the value of the vehicle.

22 A nudge of a different kind was the default enrollment into a pension. As stated in the Federal Budget of 2010, "research has shown that the key to saving is to make it automatic and simple. Under this proposal, employees will be automatically enrolled in workplace pension plans—and will be allowed to opt out if they choose. … Experts estimate that this program will dramatically increase the savings participation rate for low and middle-income workers to around 80 percent." Despite its unveiling in the budget proposal, and unlike the United Kingdom, the US Department of Labor has not pursued implementation of the initiative (OMB 2010: 37).

23 Open government was also Sunstein’s answer to the charge of paternalism sometimes leveled against his behavioral polices. He argues that making government processes, decisions, and information open and transparent will make government more efficient and “create a strong safeguard against ill-considered or ill-motivated plans” (Sunstein and Thaler 2008: 240).

24 One example that illustrates how standards of evidence empower and disempower types of expertise can be found in the March 2010 OIRA decision to return the Department of Transport’s ruling on efficiency rating requirements for replacement tires. The Department had been focused on the tests that tires should be subject to, i.e. on the physics of tire safety and on processes of test certification. OIRA’s focus was on the labeling, encouraging “clarity” of the most relevant information and “intelligibility” in use of scales. OIRA asked the Department to test and measure consumer understanding. The key to OIRA was not to improve tire testing but to optimize the interaction between sellers and consumers enhancing behavioral outcomes. OIRA justified its intervention on the rule making of the Department of Transport as simplifying the interaction between sellers and consumers (Sunstein 2010e). In this case and others, Obama-era OIRA set itself as a crucial knowledge broker pushing rule makers to be mindful of the cognitive capacities of consumers.

25 OIRA explained in its report to Congress that “disclosure requirements should be tested in advance, preferably through quasi-experimental studies, and not merely through focus group testing, which can be unreliable as a guide to actual behavior” (OIRA 2010: 40). The retiring head of the Council of Economic Advisers supported these ideas describing these tests as “pilot projects and randomized experiments” to identify useful interventions (quoted in OIRA (2011: 60) and again in OIRA (2012: 76)).

26 For example, Obama urged states to pass $10 per hour minimum wages floors in 2013 (18 plus D.C. did so), see: https://www.whitehouse.gov/raise-the-wage; and his comments on Supreme Court ruling on same sex marriage: https://www.whitehouse.gov/the-press-office/2015/06/26/remarks-president-supreme-court-decision-marriage-equality, both accessed on November 24, 2016.

27 After leaving office, Sunstein stated that “It would not be excessive to describe OIRA as, in large part, an information aggregator” (Sunstein 2013b: 1840).

28 As well as being a model of governance that has not lost advocates and that remains a plausible candidate for implementation in Western democracies. See, for example, recent developments in the UK that show that key advisers to current government take counsel from behavioral science (Cummings 2020) and that this has shaped the government’s response to the Covid-19 pandemic and determined who gets to sit in scientific advisory bodies (Yates 2020). Despite some roll backs after Obama left office, behavioral economics has also not lost its appeal in the United States. Many of Obama’s wiz kids moved west to Silicon Valley. The former chair of the SBST, Maya Shankar, became head of Behavioral Insights at Google and “The Bay Area Obama alumni network now boasts 700 members” (Kuchler 2018). More generally, the promise of data analysis and behavioral insights continues to receive attention, and one of the key architects of behavioral policy and Cass Sunstein’s co-author, Richard Thaler, was awarded the Sveriges Riksbank Prize in Economic Sciences in Memory of Alfred Nobel the year after Obama left office (2017). We are witnessing a professionalization of behavioral policymakers and analysts. A professionalization that was started and supported by the Obama White House—the SBST team, for example, initiated a working papers series that brought together behavioral science/economics research groups representing over 20 of America’ elite research universities—but has since spread elsewhere.
